# Supplementary material for: Interaction of host gene-gut microbiota in male grading of Macrobrachium rosenbergii
Source: Microbiol Spectr. 2025 Sep 16;13(10):e01290-25. doi: 10.1128/spectrum.01290-25 (PMC12502546; doi:10.1128/spectrum.01290-25)
Supplement: Supplemental Material — Figures S1 to S8; Tables S1 to S4. [file spectrum.01290-25-s0001.docx]

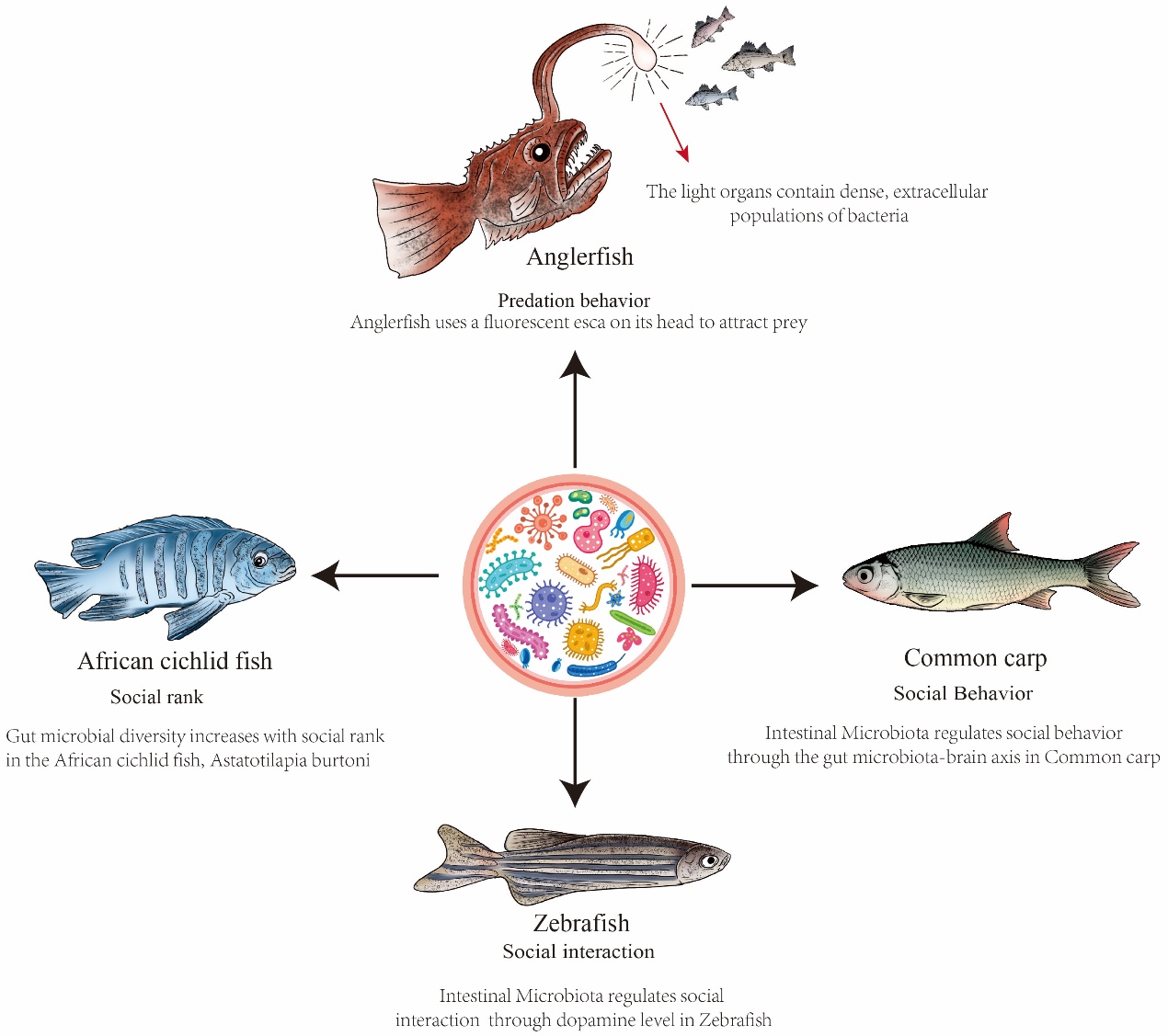
 **Fig. S1. Symbiotic microorganisms of aquatic animals regulate the social behaviors of their hosts.**

**
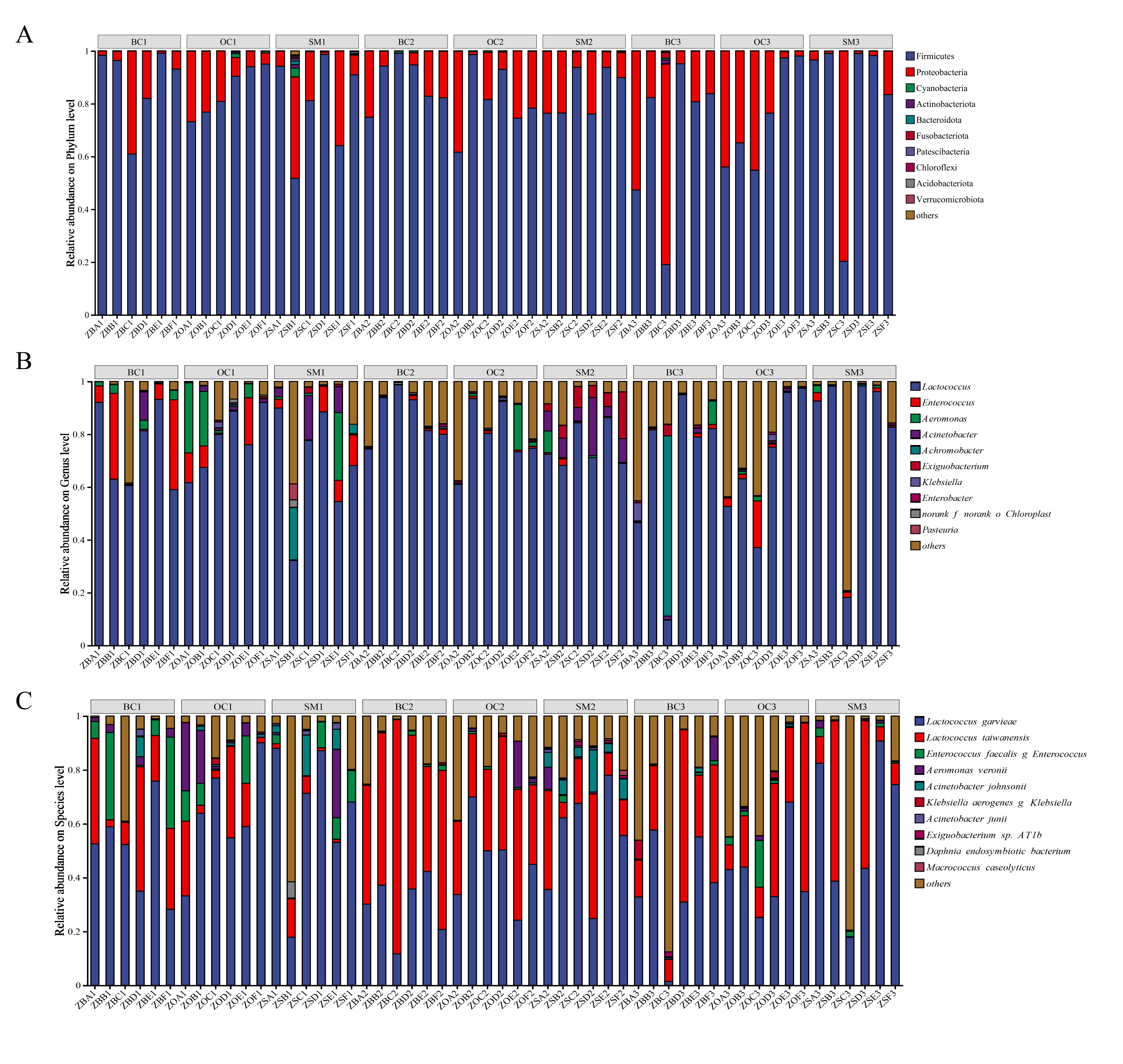
**

**Fig. S2. Abundance analysis of intestinal flora of giant freshwater prawn (GFP) at phylum, genus, and species levels.** (A), Phylum level. (B), Genus level. (C), Species level.


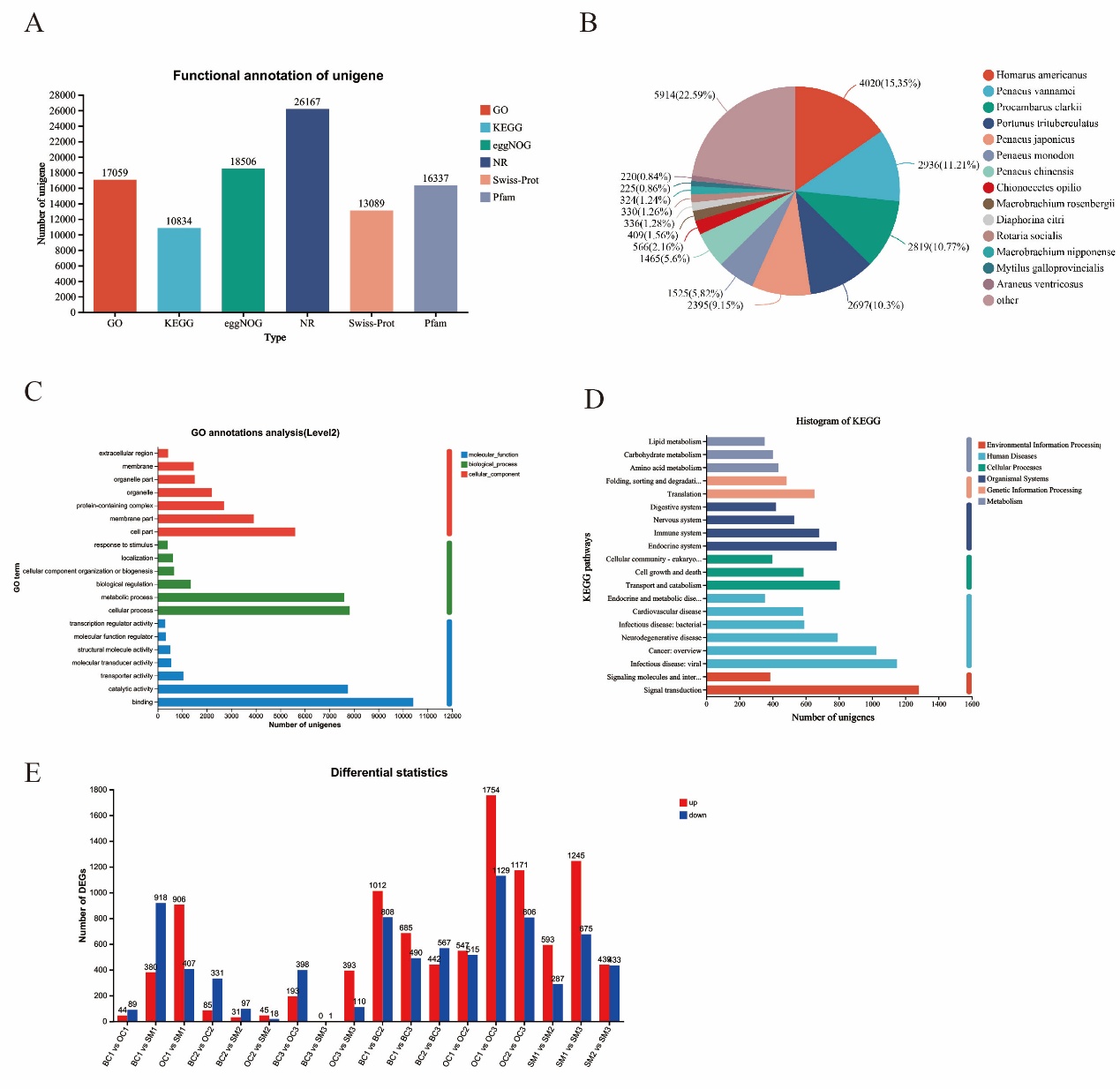
 **Fig. S3. Functional annotation of Unigenes in the gonadal transcriptome.** A, Unigenes functional annotation map; B, NR annotated species distribution pie chart; GO annotated statistical bar chart; D, KEGG pathway classification; E, statistical chart of DEGs among groups.

**
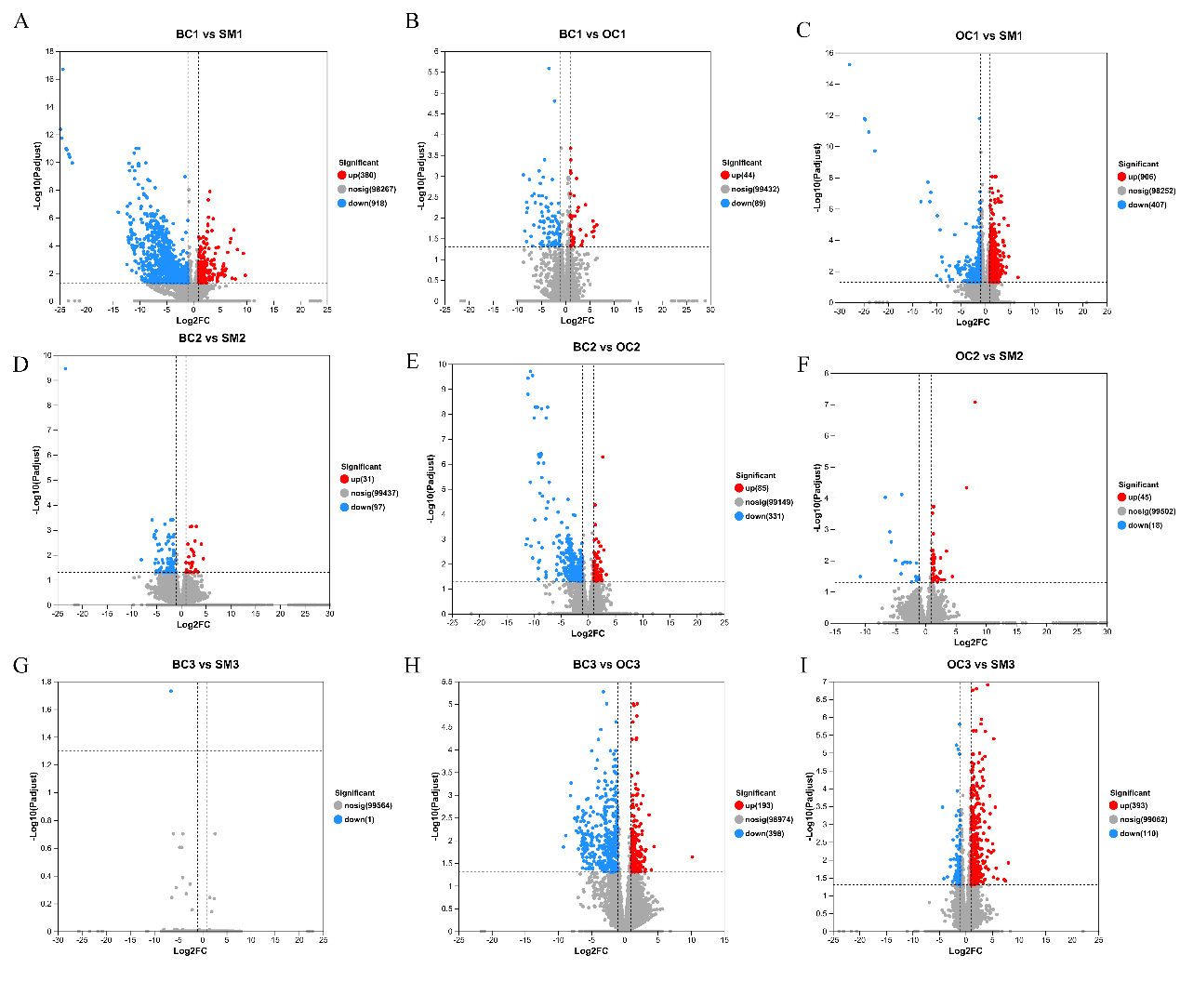
**

**Fig. S4.** **Volcano map of gene expression levels of different male grade types at different ages**

**
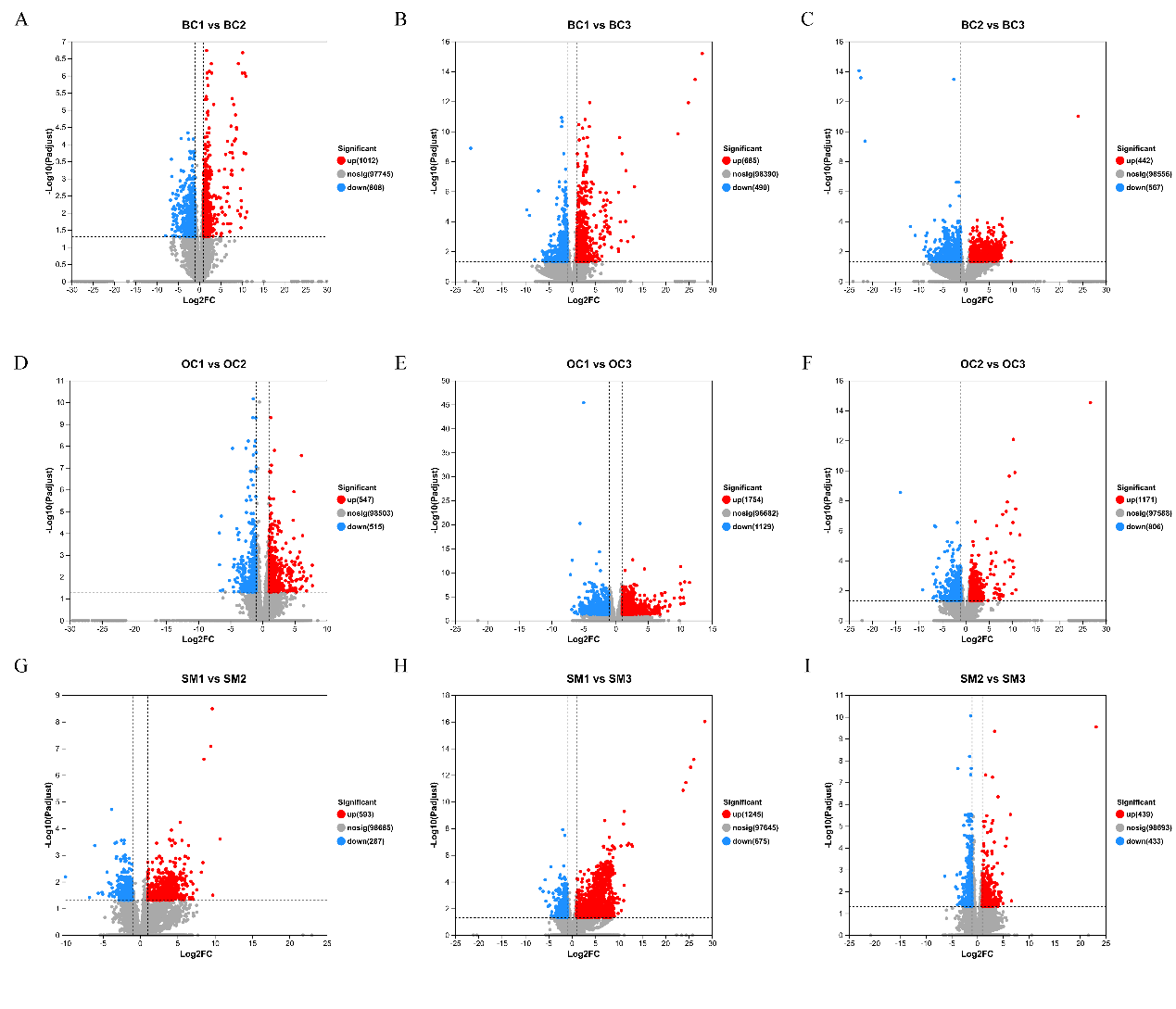
**

**Fig. S5. Volcano map of gene expression levels of same male grade types at different ages**

**
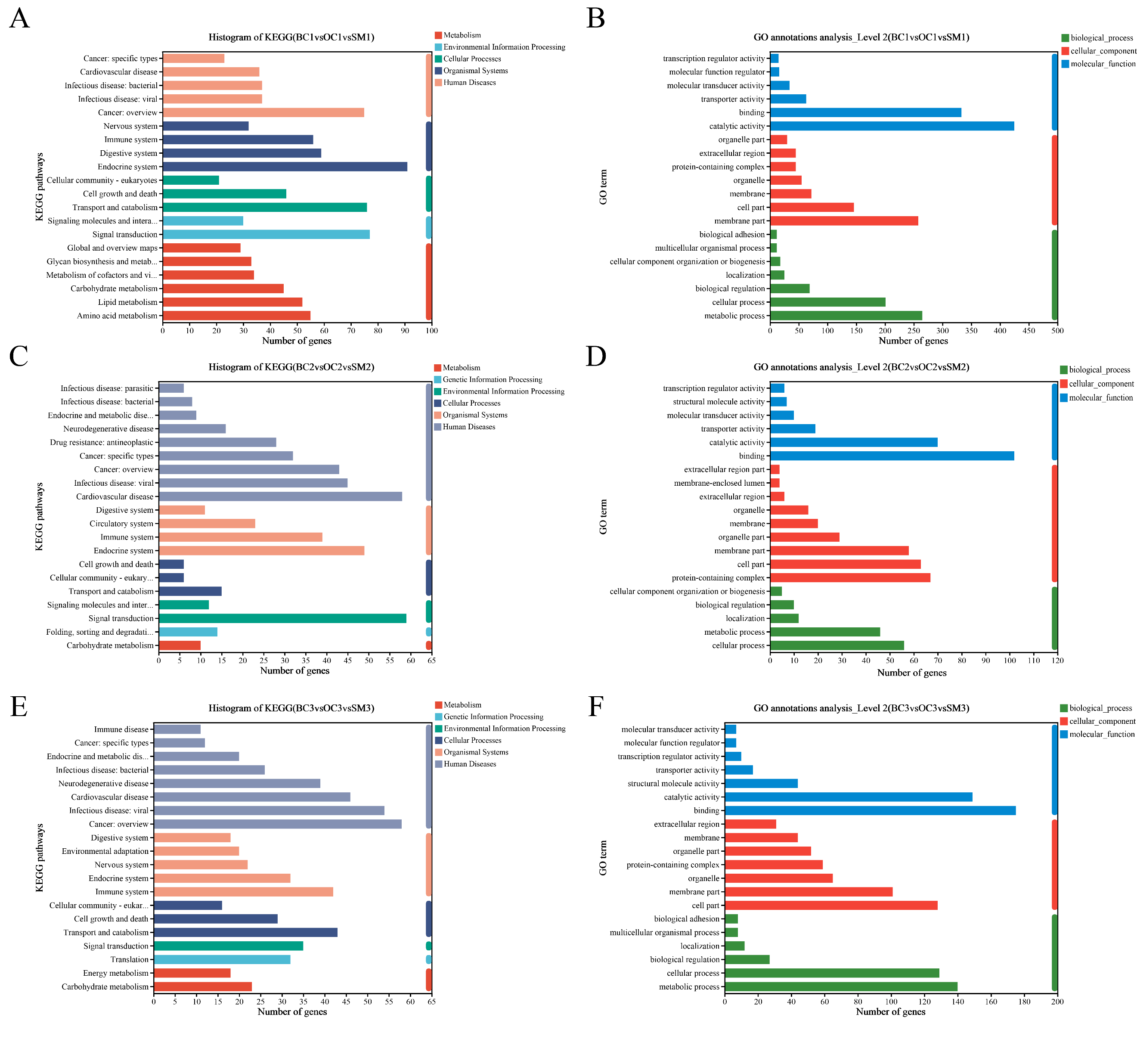
**

**Fig. S6. Gene set GO and KEGG functional annotation**

**
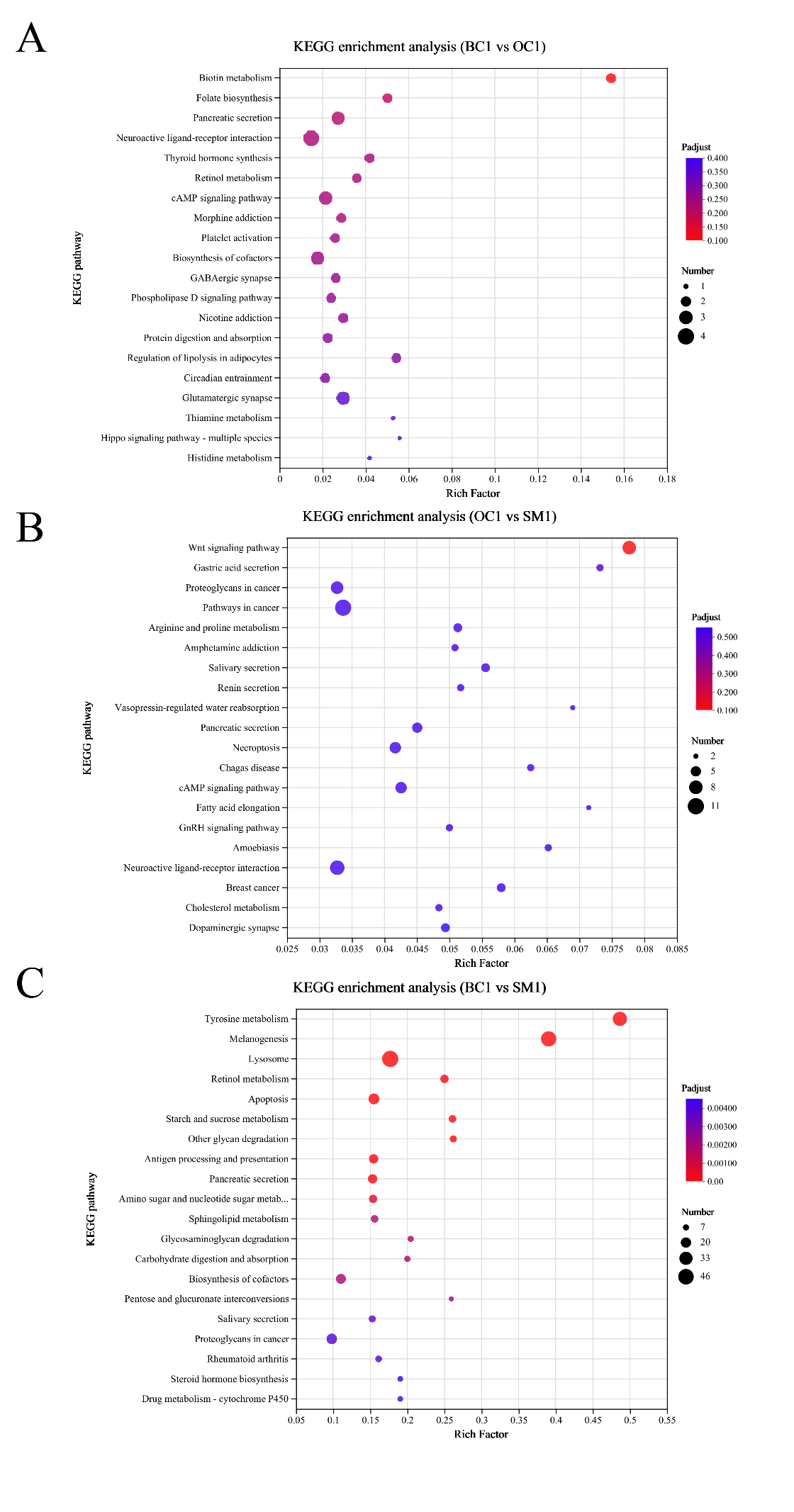
**

**
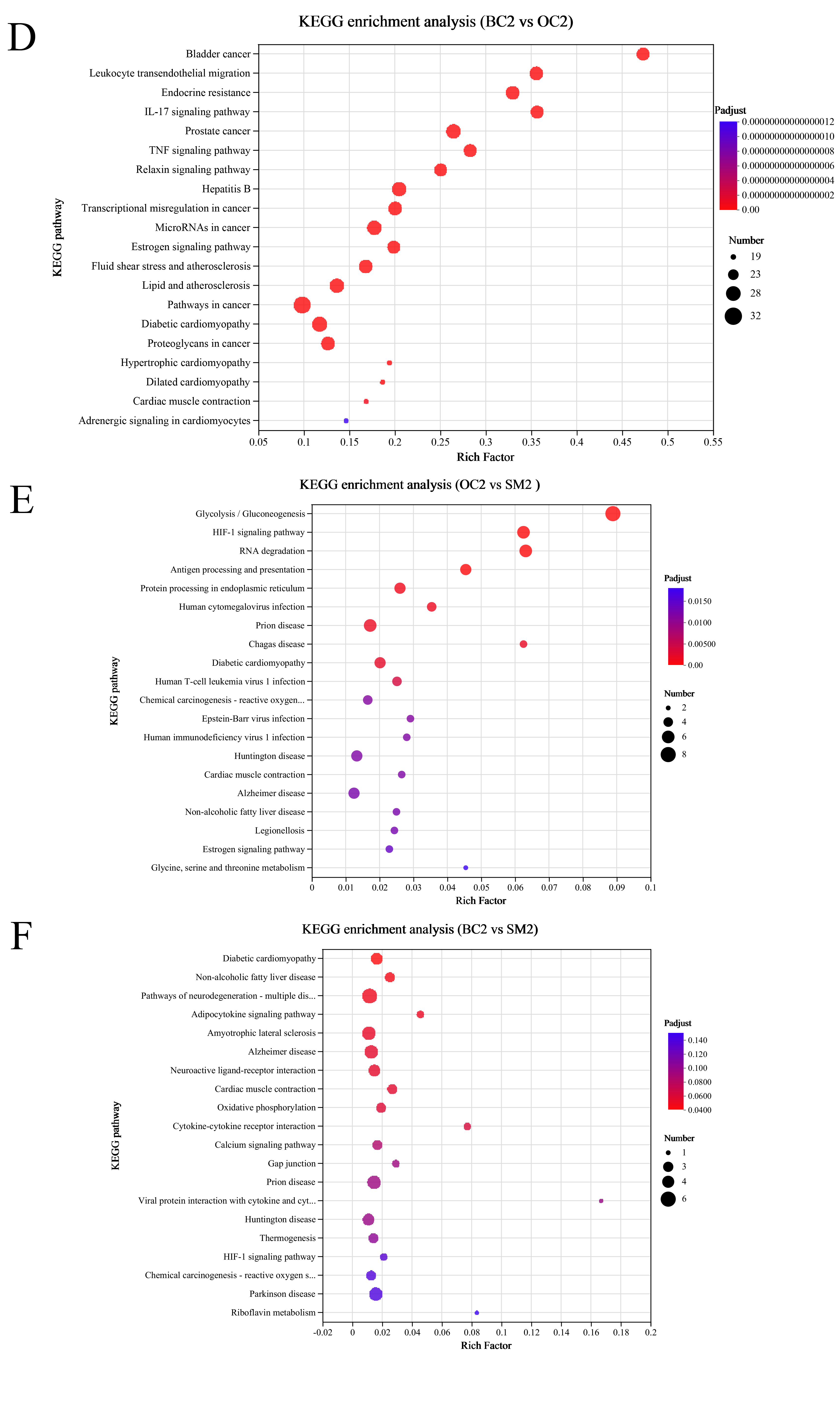

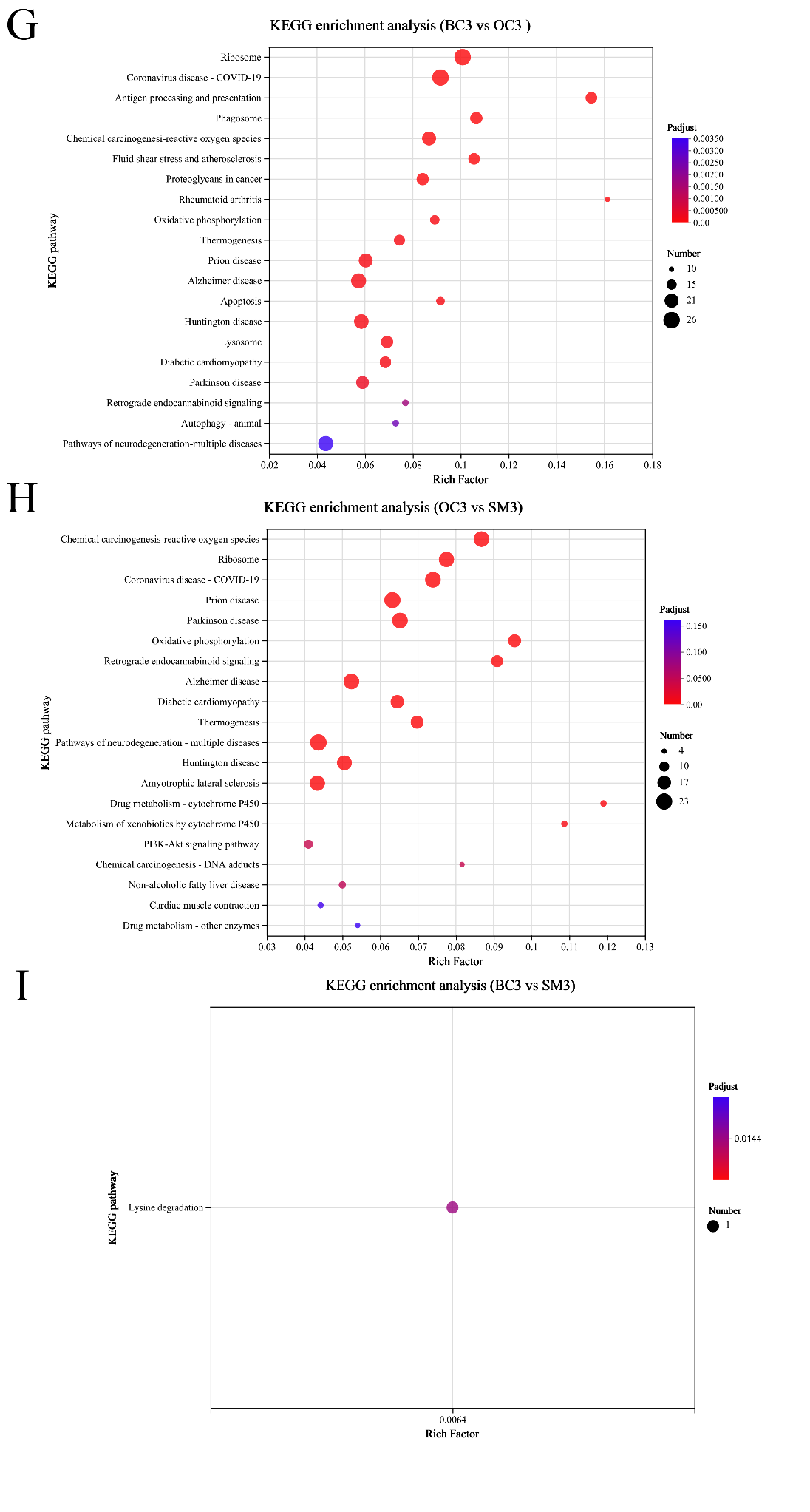
**

**Fig. S7. Enrichment analysis of DEGs KEGG pathway of male GFP at different ages.**

**
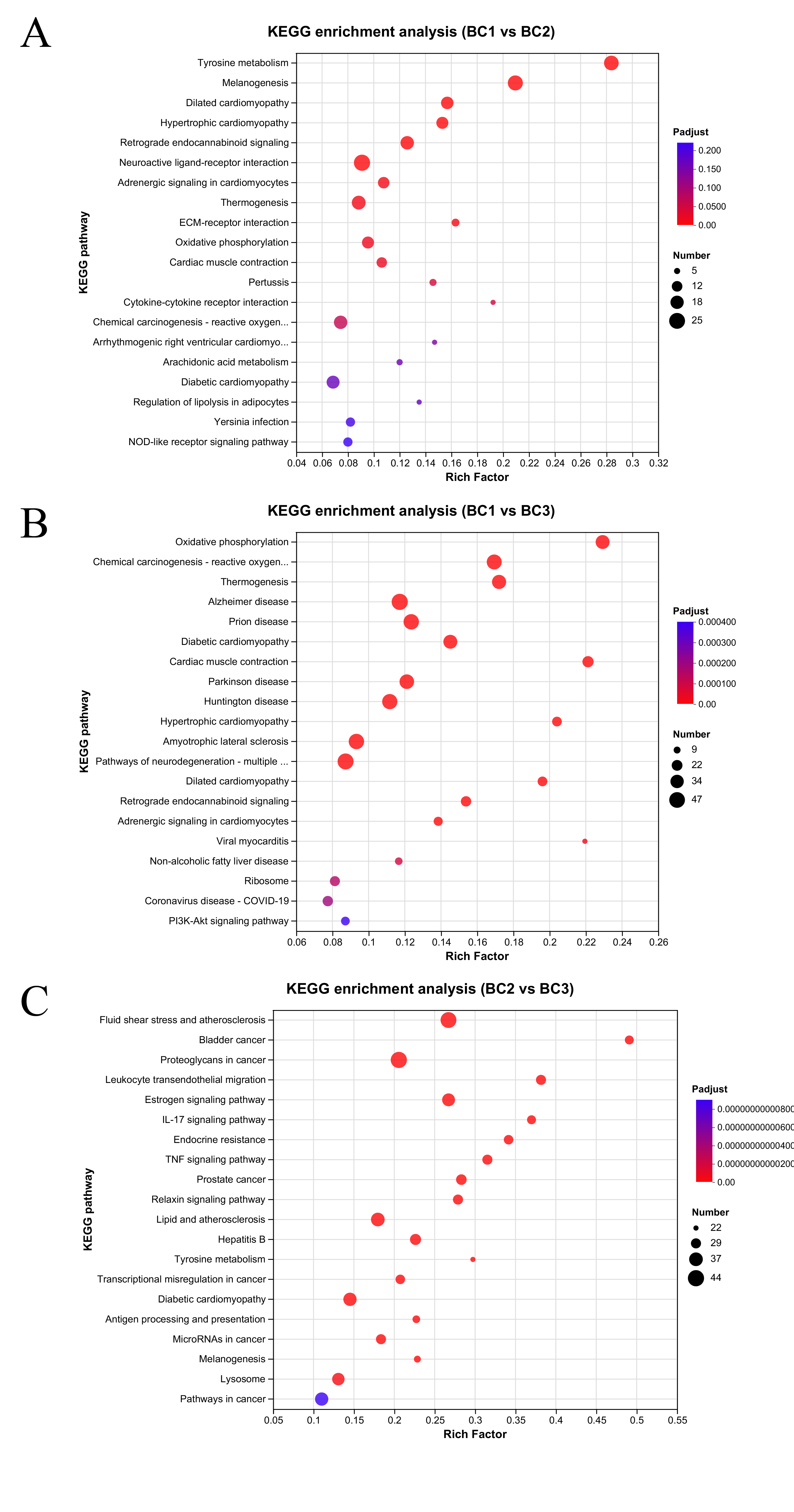

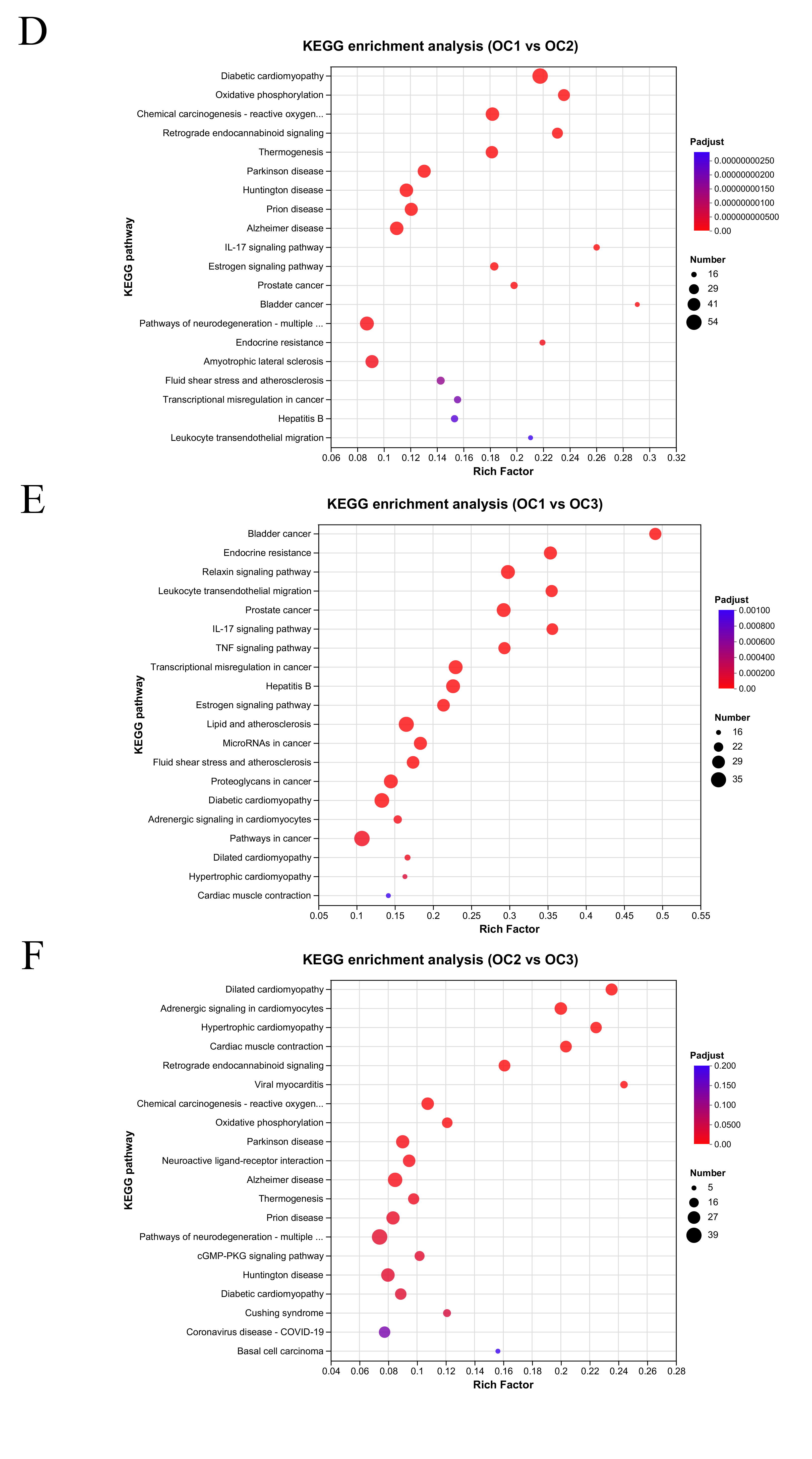

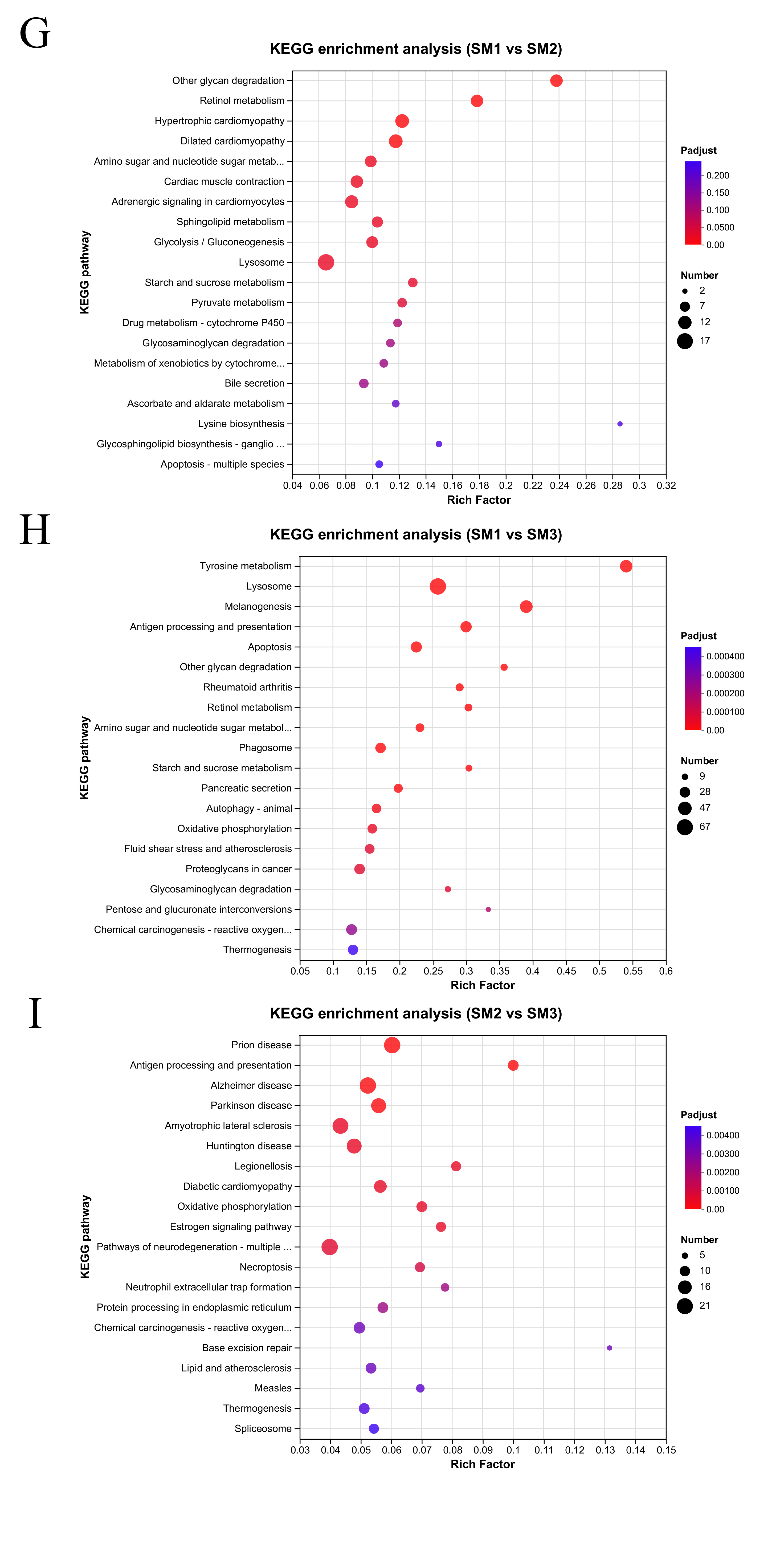
**

**Fig. S8. Enrichment analysis of DEGs KEGG pathway of the same male grade GFP at different ages**

Table S1. OTU species classification statistics

| Samples | Sequences | OUT Numbers | Phylum Numbers | Genus Numbers | Species Numbers |
| --- | --- | --- | --- | --- | --- |
| 54 | 3116208 | 755 | 25 | 391 | 539 |

Table S2. Statistics on the number of OTUs per sample

| Sample Name | OUT Numbers |
| --- | --- |
| ZBA1 | 22 |
| ZBA2 | 26 |
| ZBD3 | 26 |
| ZSC3 | 26 |
| ZBC1 | 27 |
| ZBF3 | 31 |
| ZBA3 | 35 |
| ZOE1 | 36 |
| ZBB1 | 38 |
| ZOD3 | 39 |
| ZOB3 | 41 |
| ZOA2 | 43 |
| ZOC3 | 43 |
| ZOE2 | 44 |
| ZBF1 | 47 |
| ZBE2 | 47 |
| ZOA1 | 48 |
| ZBF2 | 48 |
| ZOF2 | 50 |
| ZBC2 | 52 |
| ZBB3 | 52 |
| ZBB2 | 53 |
| ZSF2 | 53 |
| ZBE3 | 53 |
| ZOC1 | 54 |
| ZSE1 | 54 |
| ZBD2 | 54 |
| ZSD2 | 55 |
| ZOF3 | 58 |
| ZSE3 | 58 |
| ZSB2 | 59 |
| ZOA3 | 60 |
| ZOB2 | 61 |
| ZSD3 | 62 |
| ZSA2 | 65 |
| ZSC2 | 67 |
| ZOC2 | 68 |
| ZOD2 | 68 |
| ZSF3 | 70 |
| ZBC3 | 71 |
| ZBD1 | 74 |
| ZSE2 | 75 |
| ZSA3 | 76 |
| ZSB3 | 76 |
| ZBE1 | 77 |
| ZOE3 | 81 |
| ZOB1 | 85 |
| ZSA1 | 88 |
| ZSD1 | 102 |
| ZSC1 | 109 |
| ZOF1 | 135 |
| ZSF1 | 146 |
| ZOD1 | 235 |
| ZSB1 | 323 |

Table S3. Statistical table of sequencing data of all samples after data quality control

| Sample | Clean reads | Clean bases | Error rate (%) | Q20 (%) | Q30 (%) | GC Content (%) |
| --- | --- | --- | --- | --- | --- | --- |
| ZBB3 | 61850906 | 9.14E+09 | 0.0144 | 97.28 | 92.24 | 45.54 |
| ZBC3 | 57710914 | 8.59E+09 | 0.0144 | 97.26 | 92.2 | 43.96 |
| ZBD3 | 53731192 | 7.98E+09 | 0.0145 | 97.25 | 92.14 | 44.28 |
| ZOC2 | 53355040 | 7.91E+09 | 0.0146 | 97.18 | 91.98 | 44.82 |
| ZOA2 | 52517008 | 7.82E+09 | 0.0142 | 97.38 | 92.48 | 44.43 |
| ZOE2 | 51147538 | 7.58E+09 | 0.0147 | 97.15 | 91.89 | 44.53 |
| ZOB1 | 50674094 | 7.54E+09 | 0.015 | 96.97 | 91.46 | 43.68 |
| ZSF2 | 50600340 | 7.5E+09 | 0.0147 | 97.11 | 91.81 | 44.99 |
| ZOD1 | 50382960 | 7.48E+09 | 0.0148 | 97.05 | 91.69 | 42.87 |
| ZOB3 | 48380326 | 7.14E+09 | 0.0152 | 96.82 | 91.12 | 45.42 |
| ZOB2 | 48131708 | 7.14E+09 | 0.0148 | 97.09 | 91.77 | 45.44 |
| ZBF3 | 48120370 | 7.1E+09 | 0.0158 | 96.52 | 90.38 | 44.05 |
| ZOA1 | 47519908 | 7E+09 | 0.0148 | 97.08 | 91.73 | 43.92 |
| ZBA3 | 47097296 | 7E+09 | 0.0147 | 97.15 | 91.9 | 43.88 |
| ZOA3 | 46720282 | 6.91E+09 | 0.016 | 96.43 | 90.18 | 44.67 |
| ZBB1 | 46346376 | 6.91E+09 | 0.0149 | 97.02 | 91.58 | 44.78 |
| ZSC3 | 46337744 | 6.83E+09 | 0.0159 | 96.49 | 90.32 | 45.21 |
| ZSD2 | 46323324 | 6.83E+09 | 0.0148 | 97.07 | 91.71 | 43.67 |
| ZOF2 | 45744224 | 6.79E+09 | 0.0144 | 97.32 | 92.33 | 43.48 |
| ZSC1 | 45177326 | 6.71E+09 | 0.0148 | 97.08 | 91.69 | 45.83 |
| ZOE1 | 45159252 | 6.72E+09 | 0.0149 | 97.03 | 91.62 | 43.39 |
| ZSB2 | 44577936 | 6.63E+09 | 0.015 | 96.94 | 91.36 | 43.9 |
| ZOD2 | 44553274 | 6.63E+09 | 0.0147 | 97.14 | 91.86 | 44.56 |
| ZSA3 | 44450466 | 6.61E+09 | 0.015 | 96.89 | 91.4 | 44.23 |
| ZOD3 | 44395950 | 6.6E+09 | 0.0155 | 96.64 | 90.75 | 43.35 |
| ZOF3 | 44210608 | 6.56E+09 | 0.0156 | 96.63 | 90.69 | 46.8 |
| ZSE2 | 44207862 | 6.57E+09 | 0.0145 | 97.21 | 92.08 | 44.84 |
| ZBA1 | 44044582 | 6.53E+09 | 0.015 | 96.99 | 91.49 | 44.42 |
| ZSA1 | 43874122 | 6.53E+09 | 0.0146 | 97.21 | 92.03 | 46.19 |
| ZSA2 | 43873670 | 6.54E+09 | 0.0148 | 97.07 | 91.69 | 43.96 |
| ZOE3 | 43807356 | 6.52E+09 | 0.0158 | 96.51 | 90.42 | 43.48 |
| ZSE3 | 43658782 | 6.43E+09 | 0.0157 | 96.61 | 90.62 | 43.65 |
| ZBF2 | 43629776 | 6.47E+09 | 0.0148 | 97.08 | 91.7 | 44.04 |
| ZBD2 | 43599414 | 6.49E+09 | 0.0144 | 97.27 | 92.19 | 43.12 |
| ZBC1 | 43444342 | 6.46E+09 | 0.0151 | 96.91 | 91.3 | 43.98 |
| ZBF1 | 43356806 | 6.42E+09 | 0.015 | 96.94 | 91.4 | 43.25 |
| ZSF1 | 43331436 | 6.45E+09 | 0.0148 | 97.05 | 91.64 | 44.37 |
| ZOF1 | 43169912 | 6.41E+09 | 0.0148 | 97.1 | 91.75 | 45.49 |
| ZSD1 | 43035256 | 6.43E+09 | 0.0149 | 97.02 | 91.59 | 43.89 |
| ZSB1 | 42849376 | 6.35E+09 | 0.0148 | 97.07 | 91.71 | 44.94 |
| ZSF3 | 42824752 | 6.34E+09 | 0.0158 | 96.5 | 90.32 | 45.1 |
| ZBB2 | 42789696 | 6.39E+09 | 0.0148 | 97.03 | 91.64 | 42 |
| ZOC3 | 42408140 | 6.26E+09 | 0.0164 | 96.22 | 89.65 | 44.4 |
| ZBE2 | 42358250 | 6.3E+09 | 0.0149 | 97.01 | 91.57 | 43.91 |
| ZSD3 | 42344296 | 6.26E+09 | 0.0156 | 96.6 | 90.58 | 44.73 |
| ZBE1 | 42256328 | 6.3E+09 | 0.0149 | 96.99 | 91.49 | 44.1 |
| ZBE3 | 42063904 | 6.13E+09 | 0.0156 | 96.65 | 90.68 | 44.57 |
| ZSB3 | 41520656 | 6.16E+09 | 0.0161 | 96.37 | 90.02 | 44.34 |
| ZBC2 | 41317492 | 6.13E+09 | 0.015 | 96.96 | 91.43 | 43.48 |
| ZBA2 | 40987920 | 6.08E+09 | 0.0149 | 97 | 91.51 | 44.6 |
| ZSE1 | 40583210 | 6.02E+09 | 0.0149 | 97.02 | 91.56 | 45.35 |
| ZSC2 | 40461428 | 6.03E+09 | 0.015 | 96.94 | 91.4 | 42.64 |
| ZBD1 | 40349234 | 6.02E+09 | 0.0152 | 96.85 | 91.16 | 44.52 |
| ZOC1 | 40301430 | 6E+09 | 0.0149 | 97 | 91.51 | 43.65 |
| Average | 45512329 | 6.75E+09 | 0.01502 | 96.95 | 91.42 | 44.31 |

Note*:* ZB, ZO and ZS represent blue claw males (BC), orange claw males (OC), and small males (SM), respectively. Letters A-E indicate sample repetition. Numbers 1, 2, 3 indicate the age of GFP in days: 100, 110 and 120 days, respectively.

Table S4. Evaluation of sequencing data assembly optimization results

| Type | Unigene | Transcript |
| --- | --- | --- |
| Total number | 99565 | 172383 |
| Total bases | 131330804 | 232050629 |
| Largest length (bp) | 33376 | 33376 |
| Smallest length (bp) | 201 | 201 |
| Average length (bp) | 1319.05 | 1346.13 |
| N50 length (bp) | 2504 | 2646 |
| E90N50 length (bp) | 5406 | 4802 |
| GC percent (%) | 40.05 | 40.38 |
